# Supplementary material for: Mechanistic theory predicts the effects of temperature and humidity on inactivation of SARS-CoV-2 and other enveloped viruses
Source: eLife. 2021 Jul 13;10:e65902. doi: 10.7554/eLife.65902 (PMC8277363; doi:10.7554/eLife.65902)
Supplement: Transparent reporting form [file elife-65902-transrepform.pdf]

eLIFE

1st Floor
24 Hills Road
Cambridge CB2 1JP, UK

P 01223 855340
W elifesciences.org
T @elife

eLife's transparent reporting form

We encourage authors to provide detailed information *within their submission* to facilitate the interpretation and replication of experiments. Authors can upload supporting documentation to indicate the use of appropriate reporting guidelines for health-related research (see EQUATOR Network), life science research (see the BioSharing Information Resource), or the ARRIVE guidelines for reporting work involving animal research. Where applicable, authors should refer to any relevant reporting standards documents in this form.

If you have any questions, please consult our Journal Policies and/or contact us: editorial@elifesciences.org.

Sample-size estimation

- You should state whether an appropriate sample size was computed when the study was being designed
- You should state the statistical method of sample size computation and any required assumptions
- If no explicit power analysis was used, you should describe how you decided what sample (replicate) size (number) to use

Please outline where this information can be found within the submission (e.g., sections or figure legends), or explain why this information doesn't apply to your submission:

We did not do an explicit power analysis. Our statistical approach is Bayesian, without significance cutoffs, so standard frequentist power analysis does not apply. Instead, Bayesian experimental design requires obtaining sufficient data to have sufficiently certain posterior estimates of the quantities of interest. We pre-determined our experimental setup (number of replicates, number of titration wells, and timepoints to collect) based on our own prior work on SARS-CoV-2 stability (van Doremalen et al 2020, DOI:10.1056/NEJMc2004973, Fischer et al. 2020 DOI:10.1101/2020.04.11.20062018), which suggested that this would give us a sufficient range of titers over time to readily estimate the half-lives of interest to sufficient certainty. This proved to be the case.

Replicates

- You should report how often each experiment was performed
- You should include a definition of biological versus technical replication
- The data obtained should be provided and sufficient information should be provided to indicate the number of independent biological and/or technical replicates
- If you encountered any outliers, you should describe how these were handled
- Criteria for exclusion/inclusion of data should be clearly stated
- High-throughput sequence data should be uploaded before submission, with a private link for reviewers provided (these are available from both GEO and ArrayExpress)

Please outline where this information can be found within the submission (e.g., sections or figure legends), or explain why this information doesn't apply to your submission:

For each time point of each experimental condition, we used 3 biological replicates (3 separate depositions onto 3 seperate polypropylene disks, with the solution deposited drawn from the same initial stock), and for each replicate we carried out 4 replicate titrations, using dilutions from 0-fold to 7-fold. This information appears in our Methods section.

eLife Sciences Publications, Ltd is a limited liability non-profit non-stock corporation incorporated in the State of Delaware, USA, with company number 5030732, and is registered in the UK with company number FC030576 and branch number BR015634 at the address 1st Floor, 24 Hills Road, Cambridge CB2 1JP | August 2014

Statistical reporting

- Statistical analysis methods should be described and justified
- Raw data should be presented in figures whenever informative to do so (typically when N per group is less than 10)
- For each experiment, you should identify the statistical tests used, exact values of N, definitions of center, methods of multiple test correction, and dispersion and precision measures (e.g., mean, median, SD, SEM, confidence intervals; and, for the major substantive results, a measure of effect size (e.g., Pearson's r, Cohen's d)
- Report exact p-values wherever possible alongside the summary statistics and 95% confidence intervals. These should be reported for all key questions and not only when the p-value is less than 0.05.

Please outline where this information can be found within the submission (e.g., sections or figure legends), or explain why this information doesn't apply to your submission:

All statistical methods are described in brief in the Methods and at length in the Appendix. Raw individual virus titer estimates and raw evaporation data are plotted. All statistical methods are Bayesian. We use bespoke statistical models; these models are described in full mathematical detail in the Appendix, and full Stan code implementing them is provided. Full raw data is provided for statistical replication.

(For large datasets, or papers with a very large number of statistical tests, you may upload a single table file with tests, Ns, etc., with reference to sections in the manuscript.)

Group allocation

- Indicate how samples were allocated into experimental groups (in the case of clinical studies, please specify allocation to treatment method); if randomization was used, please also state if restricted randomization was applied
- Indicate if masking was used during group allocation, data collection and/or data analysis

Please outline where this information can be found within the submission (e.g., sections or figure legends), or explain why this information doesn't apply to your submission:

Virus samples used for all temperature and humidity conditions were taken from the same virus stock. This information appears in methods section.

Additional data files ("source data")

- We encourage you to upload relevant additional data files, such as numerical data that are represented as a graph in a figure, or as a summary table
- Where provided, these should be in the most useful format, and they can be uploaded as "Source data" files linked to a main figure or table
- Include model definition files including the full list of parameters used
- Include code used for data analysis (e.g., R, MatLab)
- Avoid stating that data files are "available upon request"

Please indicate the figures or tables for which source data files have been provided:

All code and data needed to reproduce all results, figures, and tables is archived on Github (https://github.com/dylanhmorris/sars-cov-2-temp-humidity/) and on Zenodo (https://doi.org/10.5281/zenodo.4093264), and licensed for reuse, with appropriate attribution/citation, under a BSD 3-Clause Revised License. An equivalent code and data availability statement appears in the manuscript.
